# Microbial Oxidation of Arsenite: Regulation, Chemotaxis, Phosphate Metabolism and Energy Generation

**DOI:** 10.3389/fmicb.2020.569282

**Published:** 2020-09-15

**Authors:** Kaixiang Shi, Qian Wang, Gejiao Wang

**Affiliations:** ^1^State Key Laboratory of Agricultural Microbiology, College of Life Science and Technology, Huazhong Agricultural University, Wuhan, China; ^2^Department of Microbiology and Immunology, Montana State University, Bozeman, MT, United States

**Keywords:** microbial arsenite oxidation, transcriptional regulation, chemotaxis, phosphate uptake, energy generation

## Abstract

Arsenic (As) is a metalloid that occurs widely in the environment. The biological oxidation of arsenite [As(III)] to arsenate [As(V)] is considered a strategy to reduce arsenic toxicity and provide energy. In recent years, research interests in microbial As(III) oxidation have been growing, and related new achievements have been revealed. This review focuses on the highlighting of the novel regulatory mechanisms of bacterial As(III) oxidation, the physiological relevance of different arsenic sensing systems and functional relationship between microbial As(III) oxidation and those of chemotaxis, phosphate uptake, carbon metabolism and energy generation. The implication to environmental bioremediation applications of As(III)-oxidizing strains, the knowledge gaps and perspectives are also discussed.

## Introduction

Arsenic (As) is a ubiquitous metalloid in the environment, and it belongs to group 15 in the periodic table, positioned directly below phosphorus (Strawn, 2018). Arsenic exists in various forms, such as inorganic trivalent arsenite [As(III)], inorganic pentavalent arsenate [As(V)], trivalent organoarsenicals, pentavalent organoarsenicals and thioarsenicals (Chen J. et al., 2015; McDermott et al., 2020). Of which, As(III) and As(V) are the most prevalent forms (Zhu et al., 2014). The toxicity of arsenic makes it to be a class I human carcinogen on the list announced by the International Agency of Research on Cancer (IARC) (Li et al., 2014; Minatel et al., 2018). Arsenic is released into the environment by both natural processes (such as volcanic eruption and parent material weathering) and the consumption of arsenic-containing products (such as chemotherapeutic drugs, insecticides and wood preservatives) (Zhu et al., 2014; Bhowmick et al., 2018). Over 200 million people are currently affected by high groundwater arsenic pollution in several areas, including Bangladesh, Brazil, Canada, China, Hungary, India, Indonesia, Mexico, Pakistan, United States and Vietnam (Chakraborti et al., 2009; Rodriguez-Lado et al., 2013; Bhowmick et al., 2018; de Souza et al., 2018). Chronic exposure to arsenic has been documented to lead to cardiovascular, diabetic, reproductive, hematological, hepatic, neurological and respiratory diseases as well as to cause bladder, lung and skin cancers (Rahman et al., 2009; Carlin et al., 2015).

Microorganisms play an important role in arsenic bioavailability, mobility and speciation in nature (Zhu et al., 2014). Microbial As(III) oxidation is considered environmental bioremediation because As(V) is less toxic and mobile than As(III) (Xiong et al., 2010; Wang et al., 2015b). To date, different genera of As(III)-oxidizing bacteria and archaea have been reported, including α*-*,β*-*,γ*-*Proteobacteria, *Thermus*, green sulfur bacteria, filamentous green non-sulfur bacteria, Crenarchaeota and Euryarchaeota (Stolz et al., 2006; Li X. et al., 2013). Furthermore, some As(III)-oxidizing bacteria use As(III) oxidation as an energy source (Oremland and Stolz, 2003; Stolz et al., 2006; Cai et al., 2009a). Chemolithoautotrophic As(III)-oxidizing bacteria, such as *Rhizobium* sp. NT-26, use As(III) as the electron donor, oxygen as the electron acceptor, and carbon dioxide-bicarbonate as the carbon source to maintain bacterial growth (Santini et al., 2000). Moreover, heterotrophic As(III)-oxidizing bacteria, such as *Hydrogenophaga* sp. N14 and *Agrobacterium tumefaciens* GW4, employ As(III) oxidation as an energy resource (vanden Hoven and Santini, 2004; Wang et al., 2015a). In recent years, the detailed regulatory mechanisms of bacterial As(III) oxidation have been revealed by our group and other researchers.

## As(III) Oxidase Catalyzes Microbial As(III) Oxidation

Microbial As(III) oxidation is catalyzed by As(III) oxidase, AioBA (Anderson et al., 1992), which is widely distributed in heterotrophic and chemolithoautotrophic microorganisms (Yan et al., 2019). AioBA was first purified from the *Alcaligenes faecalis* bacterium in 1992 (Anderson et al., 1992), and additional characteristics were identified in different As(III) oxidizers, such as *Herminiimonas arsenicoxydans* ULPAs1 and *Rhizobium* sp. NT-26 (Santini et al., 2000; Muller et al., 2003; Warelow et al., 2017; Watson et al., 2017). To date, homologs of genes encoding AioBA have been found in phylogenetically diverse strains including members of α*-*,β*-*,γ-Proteobacteria, Actinobacteria, Aquificae, Bacteroidetes, Chlorobi, Chloroflexi, Crenarchaeota, Deinococcus-Thermus, Firmicutes and Nitrospira (Yamamura and Amachi, 2014).

AioBA is a heterotetramer consisting of a large catalytic subunit (AioA) and a small subunit (AioB) (Silver and Phung, 2005). AioA is divided into four domains, in which, domain I contains a bound [3Fe-4S] cluster and a Rieske subunit and domains II-IV contain bound molybdenum (Ellis et al., 2001). AioB contains a Rieske [2Fe-2S] domain, which is homologous to cytochrome bc_1_, cytochrome b_6_f and naphthalene 1,2-dioxygenase (Ellis et al., 2001). A recent study has shown that AioBA also acts as an antimonite [Sb(III)] oxidase, indicating the complex function of AioBA (Wang et al., 2015b). Additionally, another type of As(III) oxidase, ArxA, has also been identified in a limited number of bacteria, predominantly in γ*-*Proteobacteria isolated from lakes characterized by high pH and salinity (Zargar et al., 2010). ArxA represents a distinct phylogenetic clade of As(III) oxidases and has bifunctional activity for both As(III) oxidation and As(V) reduction *in vitro* (Zargar et al., 2012; Ospino et al., 2019). Besides, trivalent forms of organoarsenicals, such as methylarsenate [MAs(V)] and roxarsone [Rox(V)], are more toxic than inorganic arsenicals, (Chen J. et al., 2015; Shi et al., 2018a). A member of the NADPH-dependent FMN oxidoreductase superfamily, ArsH, is found in many bacterial arsenic resistance operons and oxidizes trivalent organoarsenicals (Chen J. et al., 2015).

## The Regulation of As(III) Oxidation by Three-Component Signal Transduction System Aioxsr

A two-component bacterial transcriptional regulatory system was identified by Kashyap et al. (2006) in *A. tumefaciens* 5A, and additional details of AioSR have been investigated in *Rhizobium* sp. NT-26 (Sardiwal et al., 2010). The sensor kinase AioS and its cognate response regulator AioR form the two-component system together (Kashyap et al., 2006). AioS contains transmembrane helices and autokinase domain, while AioR has a helix-turn-helix domain to bind DNA (Sardiwal et al., 2010). Moreover, the phosphorylation assays in *Rhizobium* sp. NT-26 reveal that His273 is the autophosphorylation site in AioS, while Asp58, Asp13 and Asp53 compose the conserved transphosphorylation site in AioR (Sardiwal et al., 2010).

**FIGURE 1:**
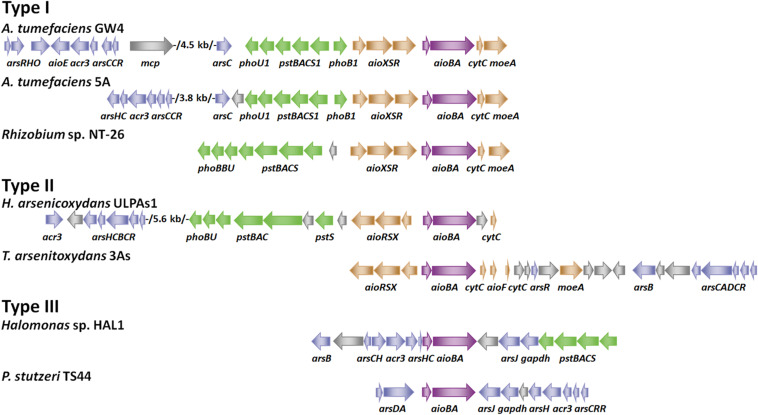
Gene arrangements of arsenic gene clusters. Arrows of different colors represent the following genes: blue for *ars* operon, green for *pst/pho* system, brown for *aioXSR*, purple for *aioBA* and gray for other genes. GenBank accession numbers are as follows: *A. tumefaciens* GW4 (NZ_AWGV00000000.1), *A. tumefaciens* 5A (NZ_AGVZ00000000.1), *Rhizobium* sp. NT-26 (NZ_FO082820.1, NZ_FO082821.1 and NZ_FO082822.1), *H. arsenicoxydans* ULPAs1 (NC_009138.1), *T. arsenitoxydans* 3As (NC_014145.1 and NC_014144.1), *Halomonas* sp. HAL1 (NZ_AGIB00000000.1) and *Pseudomonas stutzeri* TS44 (NZ_AJXE00000000.1).

Afterward, a periplasmic binding protein gene (*aioX*) is found in the upstream of bacterial *aioSR* genes, and its expression is induced by As(III) (Figure 1; Liu et al., 2012). Disruption of *aioX* inhibited the upregulation of *aioBA* genes, and consequently, As(III) oxidation (Liu et al., 2012). Without expression of a TAT-type signal peptide, purified AioX is exclusively associated with the cytoplasmic membrane, and its association constant with As(III) is 2.4 μM (Liu et al., 2012). However, site-directed mutation of Cys108 to either alanine or serine resulted in disruption of As(III) binding and *aioBA* induction, consequently causing loss of the As(III) oxidation phenotype (Liu et al., 2012). Taken together, the characterization of AioX suggests that a novel As(III)-sensing mechanism is present in a range of bacteria, which further modifies our understanding of the regulatory mechanism of As(III) oxidation from a two-component signal transduction system to a three-component signal transduction system (AioXSR). In the presence of As(III), AioX senses As(III) signals in the periplasm, thereby inducing the autophosphorylation of AioS (Kashyap et al., 2006). The phosphoryl group is then transferred from AioS to AioR, creating an active positive regulator to initiate the expression of *aioBA* genes (Kashyap et al., 2006).

According to the gene arrangements of *aioXSR* and *aioBA*, we divide As(III)-oxidizing bacteria into three types (Figure 1). Type I (*aioXSR*) is adjacent to *aioBA* with the same transcriptional direction. The three-component signal transduction system, AioXSR, is the main regulatory mechanism of As(III) oxidation in Type I typical bacteria. Type II (*aioXSR*) is adjacent to *aioBA* but has the opposite transcriptional direction. Type III has no *aioXSR* system in the bacterial genome (Cai et al., 2009b; Li et al., 2012; Li H. et al., 2013; Moinier et al., 2014; Chen F. et al., 2015; Wang et al., 2015a).

## The Regulation of As(III) Oxidation by an ArsR/SmtB Family Regulator AioF and by RpoN and RpoE2

In *Thiomonas arsenitoxydans* 3As strain, a typical Type II gene arrangement was found (Figure 1), the ArsR/SmtB family regulator AioF is encoded in the *aioBA* operon, revealing that AioF is likely involved in the regulation of *aioBA* (Moinier et al., 2014). An electrophoretic mobility shift assay (EMSA) has demonstrated that AioF binds two distinct regions upstream from *aioB* (Moinier et al., 2014). Unlike other ArsR family regulators, which bind to the genetic regulatory region to repress expression without metal(loid) and release in the presence of metal(loid) (Shi et al., 2018a), AioF specifically binds the *aioB* regulatory region in the presence of arsenic, but the interaction between AioF and DNA disappears in the absence of arsenic (Moinier et al., 2014). Additionally, the promoters of *aioB* and *aioX* are convergent, and the regulatory region of *aioB* has a putative AioR-binding site and two AioF-binding sites (Moinier et al., 2014). Thus, the regulatory mechanism in *T. arsenitoxydans* 3As strain, which consists of both AioXSR and AioF, should be a rigorous regulatory system leading to the transcription of *aioBA* in two steps. In the absence of As(III), the transcriptional *aioBA* operon is completely silent, while *aioXSR* is expressed. In the presence of As(III), As(III) is detected by AioXS, and AioR is phosphorylated, leading to transcription of *aioBA* and *aioF* at low levels (Moinier et al., 2014). In the presence of both AioR and AioF, the *aioBA* operon expression is optimal (Moinier et al., 2014).

AioSR belongs to the Sigma54 family and RpoN is the main Sigma factor for binding of the Sigma54 family regulator with the promoter (Kang et al., 2012a). Several putative RpoN-binding sites have been identified within promoters throughout the genome of *A. tumefaciens* 5A, and one putative binding site has been found in *aioBA* promoter (Kang et al., 2012a). Disruption of the RpoN-binding site in *aioBA* promoter reduced the expression of *aioBA* and bacterial As(III) oxidation (Kang et al., 2012a). In *A. tumefaciens* 5A and *H. arsenicoxydans* ULPAs1, the presence of As(III) induced the expression of *aioX*, *aioS* and *aioR* (Cleiss-Arnold et al., 2010; Liu et al., 2012). However, there is no effect on the expression of *aioX*, *aioS* or *aioR* when *Rhizobium* sp. NT-26 or *T. arsenitoxydans* 3As was grown with As(III) (Slyemi et al., 2013; Corsini et al., 2018). Interestingly, the *aioXSR* operon was upregulated in stationary phase, and the growth phase-dependent promoter RpoE2 has been reported to influence the expression of the *aioXSR* operon (Corsini et al., 2018).

## The Regulation of As(III) Oxidation by Phosphate Transport Two-Component System PhoBR

Many bacteria have two phosphate (Pi) transporters, a low affinity, high velocity Pi transporter (Pit system) and a high affinity, lower velocity Pi-specific transporter (Pst/Pho system) (Hsieh and Wanner, 2010). The Pit system serves as the primary Pi transporter when Pi is in excess, while the Pst/Pho system is activated only under low Pi concentrations (≤250 μM) (Hsieh and Wanner, 2010; Pontes and Groisman, 2018). Interestingly, genomic surveys have revealed that the two *pst/pho* systems often occur in As(III)-oxidizing bacteria (Figure 1; Li H. et al., 2013). The *pst1/pho1* system is adjacent to *aioBA* genes (Figure 1), and the *pst2/pho2* system is located elsewhere in the genome (Kang et al., 2012b; Li H. et al., 2013; Chen F. et al., 2015). The Pst/Pho system, including a Pi transport complex PstSABC and a two-component system PhoR/PhoB, is involved in Pi uptake under low Pi concentration (Qiao et al., 2019). The expression of *phoB* increased dramatically only under low Pi concentrations (≤250 μM) (Pontes and Groisman, 2018). In nature, other As(III) oxidizing bacteria were also isolated under low Pi conditions, and the efficiency of As(III) oxidation was reduced or interrupted under medium (1 mM) or high Pi conditions (>10 mM) (Kang et al., 2012b; Wang et al., 2015a, 2018). In *A. tumefaciens* strains, the disruption of *phoB1* or *pstS1* significantly decreased the transcription of *aioB*, and the efficiency of As(III) oxidation was decreased in the *phoB1* mutant (Kang et al., 2012b; Qiao et al., 2019). An EMSA assay demonstrated that PhoB1 interacted with *aioXSR* promoter to regulate bacterial As(III) oxidation (Qiao et al., 2019). Moreover, the AioSR and PhoRB signal transduction systems were capable of transphosphorylation cross-talk in *A. tumefaciens* 5A, closely integrating bacterial As(III) oxidation with the phosphate starvation response (Wang et al., 2018).

In nature, there are another type of As(III) oxidation strains (Type III) which have no *aioXSR* regulation system in the genomes (Figure 1). The bacterial As(III) oxidation regulatory mechanism in Type III strains is unique compared to those of Type I and Type II (Lin et al., 2012; Chen F. et al., 2015). In the Type III As(III)-oxidizing *Halomonas* sp. HAL1 strain, transposon mutagenesis and gene knockout assays have both shown that PhoBR affect As(III)-oxidation phenotypes in only low Pi conditions (0.1 mM, Chen F. et al., 2015). A putative Pho box was found in the *aioBA* regulatory region, and PhoB could bind the Pho box *in vivo* and *in vitro* (Chen F. et al., 2015). In conclusion, PhoBR is a regulator of some As(III)-oxidizing bacteria, implying the co-regulation of As(III) oxidation and Pi uptake under low Pi conditions.

## Cometabolism of Arsenic and Phosphorous

Generally, elements in the same group have similar chemical properties, such as silicon vs. carbon (Brzezinski, 2008) and selenium vs. sulfur (Stadtman, 1991), and substitution between these chemical analogs is common (Stadtman, 1991; Brzezinski, 2008; Rosen et al., 2011). Phosphorus is an essential nutrient for microbial biological processes. However, phosphorus is typically scarce and normally present at nanomolar levels in water and soil environments (Vieira et al., 2008). Thus, it is not surprising that As(V) can be incorporated into arsenolipids as a methylated pentavalent As-C bond and to form several types of arsenolipids in marine organisms (Edmonds and Francesconi, 1987; Dembitsky and Levitsky, 2004).

Recently, several studies have revealed that As(V) can substitute for its chemical analog Pi in bacteria (Wang et al., 2015a, 2018). Though nucleic acid and ATP may not be formed due to the instability of arsenal easter (Rosen et al., 2011), As(V) can incorporate into arsenosugars and arsenolipids with a methylated As(V)-C bond. This bond is very stable and well documented in fungi, plants, lichens and marine organisms (Dembitsky and Levitsky, 2004). More interestingly, our previous studies showed evidences of As(V) substitution of Pi in lipids in *A. tumefaciens* (Wang et al., 2015a, 2018): (1) Approximately half of As(V) was incorporated into cellular lipids in the 5A and GW4 strains under Pi-starvation and As-enriched conditions (Wang et al., 2018); (2) Compared to non-As treatment, the contents of five lipids were different with the addition of As(III), only one lipid type was increased with As(III), and this lipid contains 2-octyl-cyclopropaneoctanoic acid tail structure (Wang et al., 2015a). (3) The addition of As(V) was beneficial to the growth of As(III)-oxidizing strains 5A and GW4 in media composed of As(V):Pi ratios of 10:1, which means As(V) (1 mM for strain GW4 and 0.5 mM for strain 5A) is not toxic but beneficial to the bacteria (Wang et al., 2018).

Given that interconnection between As and Pi, it is counterintuitive that As(III)-oxidizing bacteria contain a complete and systematic regulatory link between arsenic regulation systems and the Pi signal transduction system. (1) When As(III) is present and environmental Pi is depleted, the arsenic-resistant negative regulator ArsR1 binds with As(III) and separates from the promoters of *phoB1* and *pstS1* (Garbinski et al., 2019); (2) PhoB1 is phosphorylated by the constitutively expressed histidine kinases PhoR and activates the expression of the *pst/pho* system and *aioXSR* system (Shi et al., 2018b; Rawle et al., 2019); (3) AioX senses As(III) in the environment, and self-phosphorylated AioS phosphorylates AioR to activate the expression of *aioBA*; (4) Meanwhile, phosphorylated PhoR and AioS both phosphorylate PhoB1 and AioR, which activate the *pst/pho* and *aio* systems (Wang et al., 2018; Rawle et al., 2019); (5) Expressed AioBA oxidize As(III) to As(V), PstS1 binds As(V), and the Pst1 transporter system transfers As(V) into bacterial cells. All of those evidences indicate that arsenic and Pi regulatory systems are deeply integrated and function in Pi-starvation response together (Wang et al., 2018; Rawle et al., 2019). Under Pi-starvation and arsenic-enrichment conditions, the integrated coregulation of arsenic and phosphorus metabolism is beneficial for bacteria to generate energy and use the Pi chemical analog, As(V). The consequent generation of arsenolipids may spare Pi to be recycled for nucleic acid synthesis, thereby enhancing bacterial survival potential.

## Correlation Between Arsenite Oxidation and Chemotaxis

**FIGURE 2:**
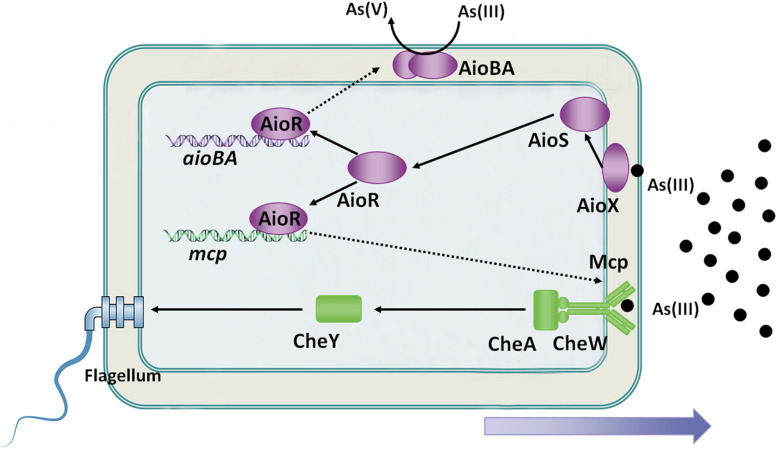
Overview of bacterial As(III) oxidation and chemotaxis. AioX binds As(III) and delivers the signal to the histidine kinase, AioS, simulating autophosphorylation of AioS. Autophosphorylated AioS then phosphorylates its cognate response regulator AioR, and subsequently AioR binds to the promoter of As(III) oxidase, which encodes *aioBA* genes, to switch on AioBA expression. Furthermore, AioR regulates expression of the As(III) chemoreceptor-encoding gene, *mcp*. Mcp detects the As(III) concentration gradient and then transports the signal to the flagellum via the chemotaxis system, CheWAY. Ultimately, the GW4 strain exhibits chemotaxis toward As(III).

Because As(III) is a toxic metalloid, it is recognized as a repellent to some microorganisms, such as *Bradyrhizobium japonicum* E10 and *Azospirillum brasilense* Az39 (Talano et al., 2013; Armendariz et al., 2015). Interestingly, As(III) is an attractant to some As(III)-oxidizing bacteria at low concentrations, such as *A. tumefaciens* GW4, *H. arsenicoxydans* ULPAs1 and *Rhizobium* sp. NT-26 (Muller et al., 2005; Andres et al., 2013; Shi et al., 2017). The As(III) chemoreceptor was first identified in *A. tumefaciens* GW4 bacterium in 2017 (Shi et al., 2017). On the genome of *A. tumefaciens* GW4, the *mcp* gene is located adjacent to the *aioXSRBA* operon, while the expression of the *mcp*, *cheA* and *cheY2* is induced by As(III) (Shi et al., 2017). Disruption of *mcp* abolished bacterial As(III) chemotaxis ability, and the purified sensing domain of Mcp specifically bound to As(III), but not to As(V) or Pi (Shi et al., 2017). Moreover, a putative AioR-binding site has been found in the regulatory region of the *mcp* gene in *A. tumefaciens* GW4, *H. arsenicoxydans* ULPAs1 and *Rhizobium* sp. NT-26 (Shi et al., 2017). The *aioR* mutant interrupted the expression of *mcp*, consequently destroying As(III) chemotaxis in *A. tumefaciens* GW4 (Shi et al., 2017). Moreover, the As(III) oxidation regulator AioR has been shown to regulate the As(III)-oxidizing bacterial chemotactic response toward As(III) (Figure 2; Shi et al., 2017).

An interesting observation derived from these studies suggests that bacterial As(III) oxidation is the essential condition of As(III) positive chemotaxis. (1) The deletion of As(III)-oxidizing genes resulted in disruption of bacterial As(III) positive chemotaxis phenotypes were observed in strains NT-26, ULPAs1 and GW4 (Koechler et al., 2010; Andres et al., 2013; Shi et al., 2017). (2) Both Mcp (association constant of 11.0 μM) and AioX (association constant of 2.4 μM) bind As(III) in the periplasm, but AioX is more sensitive to As(III) than Mcp (Liu et al., 2012; Shi et al., 2017). It indicates that AioXSR system serves as the initial As(III) response and Mcp serves as the downstream response, and such result is in agreement that bacterial As(III) chemotaxis is ultimately controlled by AioR (Figure 2; Shi et al., 2017). (3) In *H. arsenicoxydans* ULPAs1, *dnaJ* is essential for the expression of the flagellar master operon in *Escherichia coli*, and a disruption of *dnaJ* affected in both the motility and As(III) oxidation, suggesting that bacterial motility and As(III) oxidation may be co-regulated (Koechler et al., 2010). (4) Even though no clear connection was observed between AioR binding sites and motility-genes in *Rhizobium* sp. NT-26, TEM observations of the *aioR* mutant showed the presence of flagella in the early log phase of growth, suggesting that AioR may be involved in the repression of motility (Andres et al., 2013). Taken together, there is a strong correlation between bacterial As(III) oxidation and As(III) chemotaxis, and As(III) positive chemotaxis helps bacteria move toward As(III) to obtain more energy and transform As(III) to As(V), thereby enhancing bacterial survival (Shi et al., 2017).

## Physiological Relevance of Different Arsenic Sensing Systems

**FIGURE 3:**
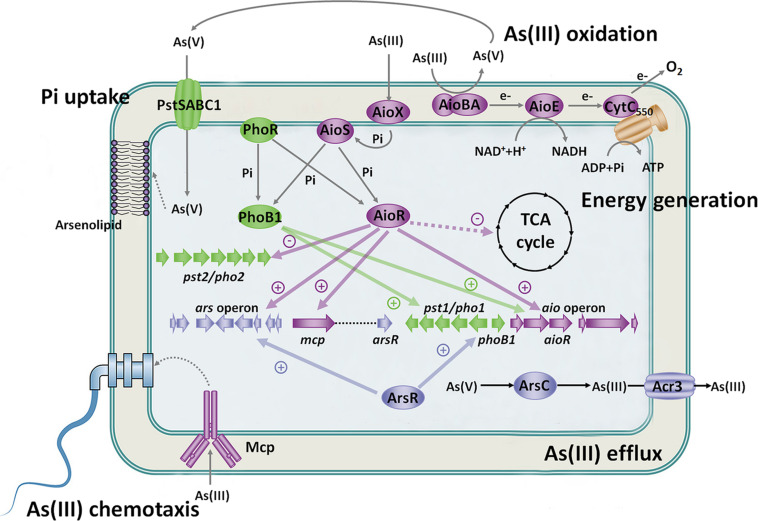
Regulatory and metabolic network related to As(III) oxidation. Under Pi-starvation and arsenic-enriched conditions, AioX and PstS sense the As(III) and As(V)/Pi signal first, and then AioS and PhoR are autophosphorylated. AioR and PhoB1 are phosphorylated by both AioS and PhoR. AioR positively regulates the expression of the As(III) oxidase AioBA and suppresses the TCA cycle so that As(III) oxidation is the main energy resource. Moreover, AioR increases the expression of the *ars* operon to enhance bacterial As(III) efflux. Moreover, ArsR activates the *pst1/pho1* system, while AioR restrains the *pst2/pho2* system to transport Pi and As(V). PhoB1 also increases the expression of the *aio* gene cluster and *pst1*/*pho1* system. The connected regulatory network between the Aio, Ars and Pst/Pho systems indicates the close relationship between As(III) oxidation, As(III) efflux and Pi uptake. Furthermore, AioR regulates the expression of *mcp* and helps bacteria move toward As(III) to produce more energy and As(V). The synthesis of arsenolipids containing As(V) may spare Pi for nucleic acid synthesis, thereby enhancing bacterial survival potential. These findings imply that As(III) oxidation is biologically part of the Pi-starvation response.

In *A. tumefaciens* strains, there are different systems that recognize arsenic (Figure 3). Aio or Mcp system sense As(III) in the periplasmic space, Pst/Pho system senses As(V) and Pi in the periplasmic space, and ArsR senses As(III) in the cytosol. The different arsenic sensing systems relate to each other via the regulatory network. Excessive accumulation of As(III) in the cytosol is harmful to cells, and the central premise of bacterial As(III) oxidation is to enable bacteria growth in As(III)-contained environment. ArsR recognizes As(III) in the cytosol and then activates the expression of the *ars* operon to excrete As(III) out of cells. Furthermore, bacterial As(III) oxidation occurs in the periplasmic space, which means that As(V) production also accumulates in the periplasmic space. Thus, it is not surprising that Aio and Mcp systems sense As(III) and Pst/Pho system senses As(V) in the periplasmic space, which is more conducive for oxidization of As(III) and acquisition of As(V). These arsenic sensing systems promote each other and enhance cell survival (Figure 3). These findings contribute significantly to our current understanding of the metabolic impact and genetic circuitry involved during As(III) exposure in microorganisms. This indicates arsenic will impact and tightly link microbial activities in global biogeochemical cycles.

## Microbial As(III) Oxidation Is Associated With Carbon Metabolism and Produces Energy

For some As(III) oxidizers, As(III) oxidation is not only a detoxification mechanism but is also related to energy generation (Santini et al., 2000; Wang et al., 2015a). This may explain why As(III)-oxidizing bacteria exhibit positive chemotaxis to As(III) (Muller et al., 2005; Andres et al., 2013; Shi et al., 2017). Some autotrophic As(III) oxidizing strains use NO_3_^–^ or O_2_ as the final electron acceptor to oxidize As(III), assisting in generation of energy for bacterial growth (Santini et al., 2000; Oremland et al., 2002). A photosynthetic As(III)-oxidizing bacterium has been reported to use As(III) as its sole photosynthetic electron donor (Kulp et al., 2008). Additionally, some heterotrophic As(III)-oxidizing bacteria, such as *Hydrogenophaga* sp. NT-14 and *A. tumefaciens* GW4, have also been reported to generate energy from As(III) oxidation (vanden Hoven and Santini, 2004; Wang et al., 2015a). Of which, *A. tumefaciens* GW4 was effective in improving the generation of ATP and NADH by As(III) oxidation (Wang et al., 2015a).

Bacterial As(III) oxidation is essentially a process of electron transport, and the electrochemical disequilibrium between reducing and oxidizing substrates is associated with energy generation (Mitchell, 1977; Ducluzeau et al., 2009). The c-type cytochrome CytC_550_ has been identified as an As(III) oxidation electron transporter in *Rhizobium* sp. NT-26 (Santini et al., 2007; Kalimuthu et al., 2014). However, the disruption of *cytC*_550_ did not completely block bacterial As(III) oxidation in *A. tumefaciens* GW4 (Wang et al., 2017). A further study identified a novel As(III) oxidation electron transporter, AioE, which belongs to the TrkA family, corresponding the generation of NADH and H^+^ (Belenky et al., 2007; Wang et al., 2009). Deletion of *aioE* resulted in a null As(III) oxidation phenotype and decreased the production of NADH, indicating that AioE is involved in As(III) oxidation electron transport (Wang et al., 2017). Furthermore, the redox potential gradient during the process of bacterial As(III) oxidation is AioBA > AioE > CytC_550_ (Wang et al., 2017). The decreased cellular concentration of ATP or NADH occurs in *cytC*_550_ or *aioE* mutants, respectively, revealing that electrons may be transferred from AioBA to AioE and finally to CytC_550_ with the generation of NADH or ATP, respectively (Wang et al., 2017).

Bacterial As(III) oxidation is associated with carbon metabolism. In the autotrophic As(III)-oxidizing strains *Thiomonas arsenivorans*, proteins involved in carbon fixation (ribulose-1,5-biphosphate carboxylase and fructose-1,6-biphosphatase) were upregulated in the presence of As(III) and the ability of carbon fixation and As(III) oxidation were both enhanced (Bryan et al., 2009). Additionally, transcriptomics and proteomics revealed that several genes and proteins involved in carbon fixation via the Calvin cycle were upregulated in *Rhizobium* sp. NT-26 grown with As(III) (Andres et al., 2013). It indicates that autotrophic As(III)-oxidizing strains may improve their capacity to fix carbon when As(III) is present (Bryan et al., 2009; Andres et al., 2013).

In the heterotrophic As(III)-oxidizing strain *H. arsenicoxydans* ULPAs1, the expression of proteins involved in the TCA cycle was upregulated in the presence of As(III) (Carapito et al., 2006; Weiss et al., 2009). An upregulated TCA cycle may be beneficial to counter the toxicity of As(III) (Carapito et al., 2006; Weiss et al., 2009). However, in *A. tumefaciens* GW4 and 5A, proteins related to the TCA cycle were downregulated, but proteins related to fermentation and As(III) oxidation processes were upregulated by As(III) (Tokmina-Lukaszewska et al., 2017; Shi et al., 2018b). Furthermore, AioR has been shown to negatively regulate the 5-dehydro-4-deoxyglucarate dehydratase encoding *kdgD*, and *kdgD* uses 5-dehydro-4-deoxy-D-glucarate as a substrate to produce 2,5-dioxopentanoate, which enters the TCA cycle (Jeffcoat et al., 1969; Shi et al., 2018b). These findings indicate that As(III) oxidation and fermentation are the main energy resources that directly contribute to energy generation in some heterotrophic As(III)-oxidizing bacteria (Shi et al., 2018b). In conclusion, the integrated relationship between As(III) oxidation and carbon metabolism may enhance bacterial survival potential under an oxygen-deficient, Pi-starvation and As-enriched environment.

## Environmental Bioremediation Applications of As(III)-Oxidizing Strains

As(III)-oxidizing microorganisms are potential candidates for bioremediation of arsenic contaminated environments due to the ability to oxidize As(III) to As(V) (Marinho et al., 2019). Apart from directly changing the chemical forms of arsenic compounds by microbial As(III) oxidation process, As(III)-oxidizing bacteria can also enhance the arsenic uptake in arsenic accumulating plants. For example, the presence of *Ensifer* sp. M14 strain in soil contributed to the increase of both growth and arsenic-uptake efficiency in alfalfa (*Medicago sativa* L., TANGO type) (Debiec-Andrzejewska et al., 2020). Compared to non-bioaugmented soil, the biomass of plants increased by about 60%, while the arsenic accumulation by alfalfa increased more than twice after 40 days (Debiec-Andrzejewska et al., 2020).

Microorganisms promote the interaction of arsenic and iron. In nature, the presence, enrichment and migration of arsenic is often associated with iron-containing minerals and As(III)-oxidizing microorganisms are used as biological reagents in combination with iron for the treatment of arsenic contamination (Karn et al., 2017; Yang et al., 2017). The combination of an As(III)-oxidizing bacterium *Brevibacterium* sp. YZ-1 and biogenic schwertmannite could immobilize arsenic in the highly arsenic contaminated soil (Yang et al., 2017). Combining an As(III)-oxidizing bacterium XS4 with FeCl_3_ also significantly stabilized arsenic in soil (Karn et al., 2017). Simultaneous removal of As(III) and was achieved by the immobilized *Enterobacter* strain in combination with FeCl_3_ and Ca(OH)_2_ (Shi et al., 2020). In addition, iron-oxidizing bacteria are also important in the oxidation of As(III). For instance, the inoculation of three arsenic resistant iron-oxidizing bacteria (*Bacillus* sp. T2, *Pseudomonas* sp. Yangling I4 and *Bacillus* sp. TF1-3) enhanced the formation of iron plaque and then decreased arsenic amount in brown rice (Xiao et al., 2020). Taken together, combining As(III)-oxidizing bacteria with chemical treatments are promising strategies for the removal or immobilization of arsenic from contaminated environment.

## Conclusion, Knowledge Gaps and Perspectives

The concentration of Pi is usually low in the environment, and microorganisms can oxidize As(III) in low Pi concentration environments. Therefore, it is not unusual that relationships between bacterial As(III) oxidation, chemotaxis, Pi transport and energy metabolism appear under Pi-starvation conditions. Here, we highlight microbial survival strategies under Pi-limited and As(III)-enriched conditions (Figure 3). As(III)-oxidizing bacteria utilize As(III) oxidation to generate energy and detoxify As(III). At a certain level of As(III), bacteria activate the As(III) positive chemotaxis system to acquire As(III) and perform As(III) oxidation. Meanwhile, the *pst1/pho1* system is upregulated to simultaneously acquire Pi and the As(III) oxidation product As(V), and As(V) may be incorporated into specific biomolecules, such as arsenolipids and arsenoproteins. The coregulation of bacterial arsenic and phosphorus metabolism benefits bacteria to recycle Pi for nucleic acid synthesis. Furthermore, the As(III) oxidation regulator AioR controls multiple As(III) resistance mechanisms to endow bacteria with strong As(III) resistance. Bacterial As(III) oxidation, chemotaxis, Pi transport and energy metabolisms synergistically benefit the survival of As(III)-oxidizing bacteria under Pi-limited and arsenic-riched conditions.

Recent literatures and our published works provide great insights for the understanding of microbial arsenite oxidation mechanisms. However, knowledge gaps still exist and more questions need to be resolved in the future. (1) Arsenic is found in bacterial lipids under Pi-limited conditions. However, the understanding of As(V) uptake and whether As(V) is incorporated in arsenolipids or replaces Pi in arsenolipids remain unknown. (2) In marine organisms, the As(III) S-adenosylmethionine methyltransferase ArsM has been reported to catalyze As(V) and other molecules to form arsenolipids (Qin et al., 2006). However, no recognizable *arsM* homolog has been identified in the *A. tumefaciens* GW4 and 5A strains, implying that arsenolipids originate from a different biosynthesis strategy in bacteria. (3) Genomic surveys have shown that genes related to the phosphonate transport system *(phn)* are often adjacent to *aioBA* genes in some As(III)-oxidizing bacteria (Manav et al., 2018). However, it remains unclear whether the Phn system is involved in the metabolism of arsenolipids. (4) As(III) oxidation regulators not only regulate As(III) oxidation but also affect multiple bacterial metabolic pathways. However, the regulatory network requires further study to clarify the biological significance of bacterial As(III) oxidation. Additionally, microbial As(III) oxidation is mainly investigated in bacteria and archaea. However, no As(III) oxidase demonstrates conserved homology in fungi (Li et al., 2014), suggesting that As(III) oxidation in fungi still needs to be investigated. (5) The application of As(III)-oxidizing microorganisms is only in the preliminary stage and the interactions among microbes, soils and plants need to be further investigated.

## Author Contributions

GW performed the study conceptualization and supervision. KS performed the literature search and wrote the original draft. KS, QW, and GW carried out the critical discussion, writing, reviewing, and editing. All authors read and approved the final manuscript.

## Conflict of Interest

The authors declare that the research was conducted in the absence of any commercial or financial relationships that could be construed as a potential conflict of interest.
